# Accidental neck and chest penetration by a metal sliver derived from an axe for wood chopping: a case report

**DOI:** 10.1186/s13256-019-2184-7

**Published:** 2019-08-17

**Authors:** Roberto Corzani, Lisa De Leonibus, Luca Luzzi, Marco Ghisalberti, Fabiola Meniconi, Tommaso Ligabue, Piero Paladini

**Affiliations:** University Hospital of Siena, Strada di Renaccio 43/B, 53100 Siena, Italy

**Keywords:** Chest trauma, Neck trauma, Penetrating trauma, Foreign body

## Abstract

**Background:**

Penetrating neck and chest trauma is a very common entity in emergency medicine that usually requires surgical treatment. Our case report illustrates the case of a 27-year-old Arabian man with hemopneumothorax associated with pneumomediastinum due to an unusual occupational injury.

**Case presentation:**

A metal sliver, coming from an axe using for wood chopping, penetrated the neck of a 27-year-old Arabian man in the left supraclavicular region mimicking a gun bullet; the entrance hole was at the left pleural dome where the sliver had just penetrated the apex of the lung passing through the upper lobe of his left lung creating an exit wound in the dorsal segment of the same lobe arriving in the posterior thoracic wall. Biportal video-assisted thoracic surgery was performed to remove blood clots and the unusual foreign body.

**Conclusion:**

In the literature, there are several case series about this topic, with some of them reporting unusual foreign bodies that lead to penetrating trauma. However, to the best of our knowledge, no cases like the one we have reported are described in the current literature.

## Background

Penetrating neck and chest trauma is a very common entity in emergency medicine that usually requires surgical treatment. In the literature, there are several documented case series about this topic with some of them reporting penetrating trauma due to unusual foreign bodies. Our case report illustrates the case of a 27-year-old man with hemopneumothorax associated with pneumomediastinum. This clinical scenario was due to an accidental occupational injury, which consisted of a penetrating trauma by a metal sliver derived from the metallic part of an axe used for wood chopping. To the best of our knowledge, no similar cases are described in the current literature.

## Clinical presentation

A 27-year-old Arabian man working as a pizza maker presented to the emergency department of Isola d’Elba Hospital 1 hour after an occupational hazard occurred while he was making firewood with an axe for the wood oven of his restaurant. He stated that a metal sliver, coming from the axe using for wood chopping, penetrated his neck in the left supraclavicular region. On admission he was fully conscious, oriented, and hemodynamically stable. He did not refer a significant past medical history. He lives with his parents. He denied tobacco, alcohol, or illicit drug use. There was no family history of stroke or vascular disease. His vital parameters were normal: his pulse rate was 70 beats per minute (bpm), his blood pressure was 140/80 mmHg, peripheral blood oxygenation was 95% without oxygen therapy, and corporeal temperature was 36.3 °C. He also did not demonstrate any clinical symptoms such as chest pain or dyspnea: respiratory excursions were full and symmetrical, lungs resonant to percussion, and vesicular breath sound was reduced in left basal field; no rales, rhonchi, wheezes, or rubs were present; vocal and tactile fremitus was reduced in the same field. The results of a cardiovascular examination were: no abnormal heaves or lifts; no trill; regular rate and rhythm; no extra sounds or murmurs. At the level of his abdomen: no abnormal tympany was detected, with normal bowel sounds; superficial and deep palpation did not reveal any organomegaly or masses; no evidence of direct or rebound tenderness, rigidity, or guarding; liver and spleen appeared to be of normal size. At a neurological examination, he was alert and attentive. His speech was clear and fluent with good repetition, comprehension, and naming. There was no pronator drift of outstretched arms. His muscle bulk and tone were normal. Strength was full bilaterally. His reflexes were 2+ and symmetric at the biceps, triceps, knees, and ankles. Plantar responses were flexor. Light touch, pinprick, position sense, and vibration sense were intact in fingers and toes. Rapid alternating movements and fine finger movements were intact. There was no dysmetria on finger-to-nose and heel-knee-shin. There were no abnormal or extraneous movements. Romberg sign was absent.

**Fig. 1:**
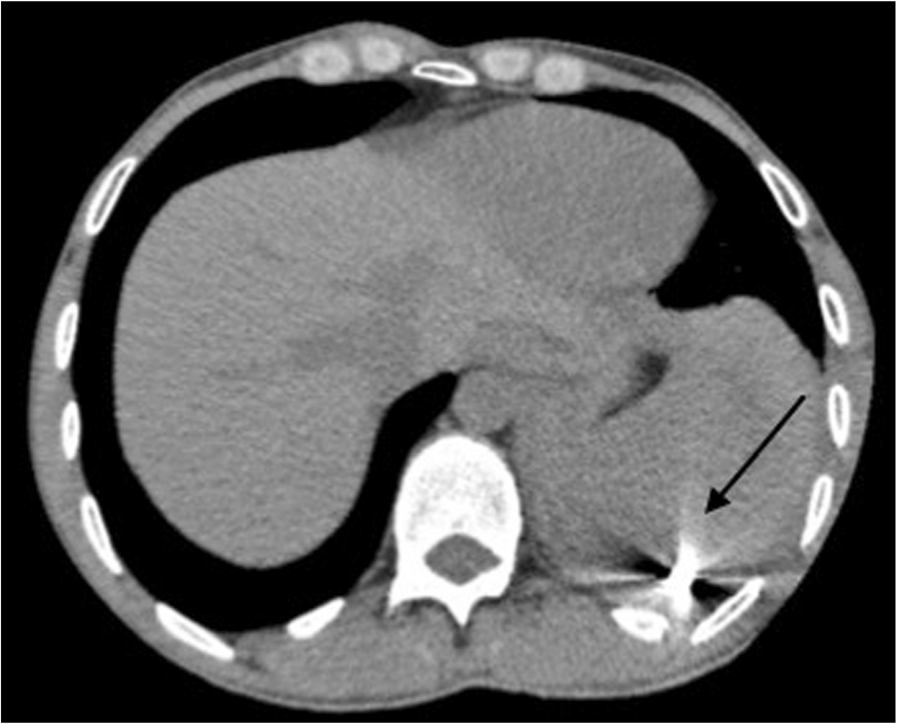
Chest computed tomography scan: evidence of a metal foreign body in the left chest cavity above the ipsilateral hemidiaphragm (the arrow indicates the metal foreign body)

**Fig. 2:**
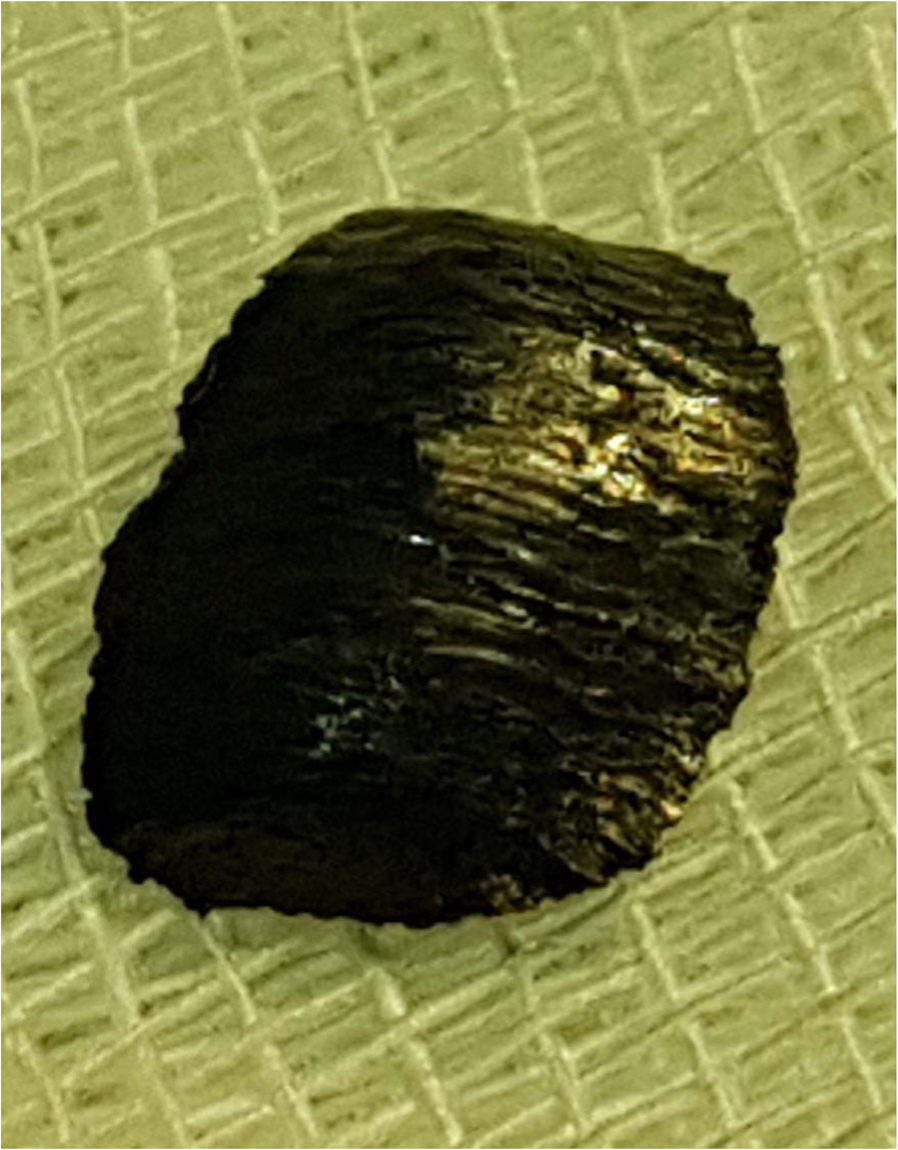
Foreign body: 1.5 cm pointed, sharp metal sliver

**Fig. 3:**
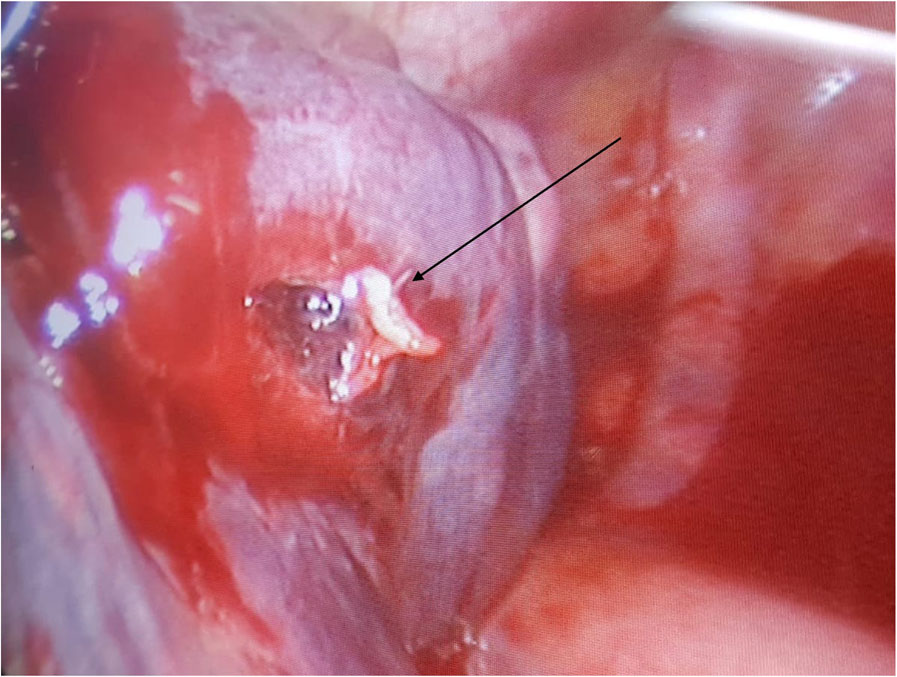
Lung injury: entrance hole at the left pleural dome (the arrow indicates the entrance hole)

**Fig. 4:**
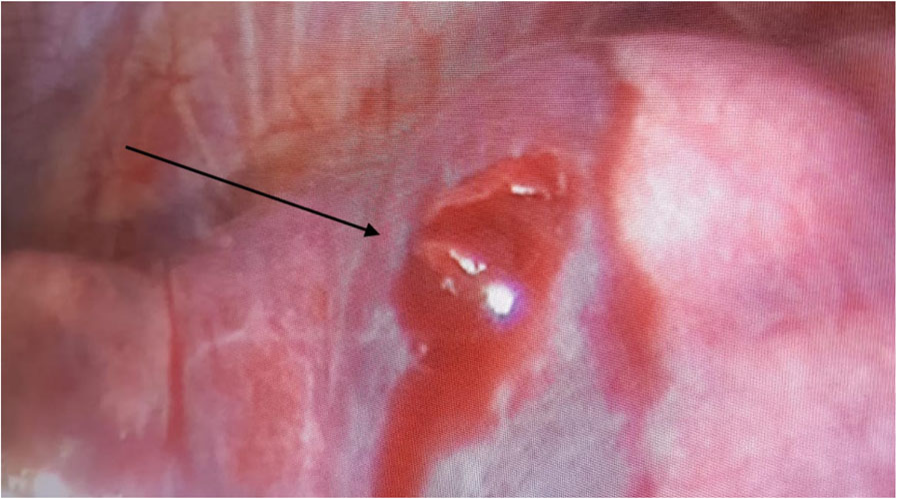
Lung injury: exit wound in the dorsal segment of the left upper lobe (the arrow indicates the exit wound)

On the basis of the collected anamnestic data, he underwent a computed tomography (CT) scan that showed left hemopneumothorax, pneumomediastinum, and subcutaneous emphysema, without any signs of active bleeding. Another important radiological finding was the evidence of a metal foreign body in the left side of his chest cavity above the ipsilateral hemidiaphragm (Fig. 1). No cervical structural injuries were detected upon radiological examination. A tube was placed in the left side of his chest with drainage of approximately 1000 mL of blood. He was then transferred by helicopter to our department in the Siena Hospital, tertiary trauma center, for the surgical removal of the foreign body. He was immediately brought to the operating room where he was intubated with a double-lumen endotracheal tube. Biportal video-assisted thoracic surgery (VATS) was performed: a 10 mm camera port was placed on the IX intercostal space, midaxillary line, and a 5 mm working port was made on the V intercostal space on the site of the chest tube, which was previously inserted. Left-sided VATS showed persistent hemothorax (600 mL) with blood clots that were removed. During the chest cavity inspection we found the foreign body, visible on the CT scan, in the left costodiaphragmatic angle. It consisted of a 1.5 cm pointed, sharp metal sliver (Fig. 2), entering his supraclavicular region. We concluded that the sliver narrowly missed the subclavian vessels, mimicking a gun bullet. In fact, while exploring his chest cavity, we noticed the entrance hole at the left pleural dome (Fig. 3) where the sliver had just penetrated the apex of the lung passing through the upper lobe of his left lung creating an exit wound in the dorsal segment of the same lobe (Fig. 4). The sliver finally arrived in the posterior thoracic wall where we found a pleural injury. We performed accurate hemostasis of the minimally bleeding sites of his chest wall and lung parenchyma by electrocautery. After a hydrostatic test that showed no air leakage from his lung we decided not to treat the parenchymal wounds. He also underwent multiple lavages of his chest cavity with the disinfectant solution Betadine (povidone-iodine), with a final instillation of a topical antibiotic (gentamicin). At the end, a chest tube inserted through the lower access was attached to a Pleur-evac® (a chest drainage system). Total operative time was 70 minutes.

During his postoperative course, no signs of bleeding or air leakage were detected. Laboratory tests did not show signs of infection or anemia: white blood cells (WBCs) 9260 cells/mcL, red blood cells (RBCs) 4.15 million cells/mcL, hemoglobin (Hb) 11.9 gm/dL, and hematocrit (HCT) 35.9% 257,000 platelets/mcL. Other laboratory analyses showed: creatinine 0.89 mg/dL, alanine aminotransferase (ALT) 32 units/L, aspartate aminotransferase (AST) 26 units/L, and bilirubin 0.6 mg/dL. Control chest X-rays showed full lung re-expansion without pleural or parenchymal pathological findings. Medical treatment consisted of amoxicillin/clavulanic acid 1 gr twice a day, paracetamol 1 gr 3 times a day, and ketorolac 30 mg according to visual analog scale (VAS) as painkillers, and lansoprazole 30 mg once a day and enoxaparin 4000 UI once a day. He was discharged on the third postoperative day.

At 6-month follow-up he underwent a thoracic CT scan that did not show any pathological findings.

## Discussion

Penetrating neck and chest trauma is a very common entity in emergency medicine that usually requires surgical treatment. Our paper illustrates the case of a 27-year-old man with hemopneumothorax associated with pneumomediastinum due to an unusual occupational injury consisting of a penetrating neck wound by a metal sliver, coming from an axe using for wood chopping. To the best of our knowledge, no similar cases were reported previously in literature.

Penetrating trauma requires immediate life-saving maneuvers, rapid transportation to a tertiary trauma center, appropriate intensive care, targeted examination with serial reassessments in the emergency department, prompt diagnosis, and surgical intervention by a multidisciplinary team. Penetrating trauma can result from occupational hazards, falls, vehicular or traffic accidents, and self-inflicted injuries. Sometimes, it may be associated with foreign body retainment, such as shell fragments, bullets, shrapnel, pieces of clothing, and bone fragments [1]. These cases can be complicated by infections, sepsis, migrating foreign bodies that may be embolic, signs of lead poisoning, lung contusion or hematoma, bone fractures, hemothorax or hemopericardium, and pneumothorax or vascular injuries. In the literature, there are several case series about this topic, with some of them reporting unusual foreign bodies that lead to penetrating trauma. For example, Laurent *et al.* and Mahjoubi *et al*. described a whiptail stingray injury causing, respectively, tension pneumothorax and hemopneumothorax associated with a tracheal wound [2, 3]. Another interesting case series was reported by Ndiaye *et al.* that described five cases of penetrating chest wounds caused by weapons made from swordfish swords [4]. Tang *et al*. presented the case report of a construction worker who fell from a height at a construction site, leading to polytrauma from iron rods penetrating through the thorax and head [5]. Another example was described by Wan and Kefaloyannis who documented a penetrating chest injury caused by a self-inflicted crossbow arrow traversing the hemithorax [6]. Finally, Kawaguchi *et al*. illustrated a male with a penetrating pulmonary artery injury due to an ice pick [7].

## Conclusions

In most of the aforementioned reports the authors described surgical intervention with a minimally invasive technique.

VATS is currently a safe and feasible alternative to thoracotomy in the management of chest trauma especially for hemodynamically stable patients. In our case, the use of the thoracoscope allowed an excellent view of the lung apex and the pleural dome, as well as the costodiaphragmatic angle. In accordance with the literature, we confirm the necessity for the use of surgical removal in the case of a retained foreign body associated with penetrating chest trauma. VATS is a valid surgical approach, although we emphasize the crucial importance of appropriate patient management in the perioperative period by a multidisciplinary team.

## Data Availability

The datasets used and/or analyzed during the current study are available from the corresponding author on reasonable request.

## Abbreviations

CT: Computed tomography
VATS: Video-assisted thoracic surgery
